# Dysregulated tRNA-derived fragments impair fatty acid metabolism in intrahepatic cholestasis of pregnancy

**DOI:** 10.3389/fmed.2025.1630677

**Published:** 2025-07-31

**Authors:** Lian Yang, Xia Yu, Shimao Zhang, Jinzhu Fu, Mengjun Luo, Wei Shen, Cheng Huang, Xiao Yang

**Affiliations:** ^1^Department of Clinical Laboratory, Chengdu Women's and Children’s Central Hospital, School of Medicine, University of Electronic Science and Technology of China, Chengdu, China; ^2^Department of Obstetrics, Chengdu Women's and Children's Central Hospital, School of Medicine, University of Electronic Science and Technology of China, Chengdu, China; ^3^Chengdu First People's Hospital, Chengdu, China

**Keywords:** intrahepatic cholestasis of pregnancy (ICP), tRNA-derived small RNAs (tsRNAs), tRNA-derived fragments (tRFs), fatty acid, total bile acid (TBA)

## Abstract

**Objective:**

Intrahepatic cholestasis of pregnancy (ICP) is a common obstetric complication that occurs predominantly in the mid-to-late gestational period, but its exact etiology remains unclear. Recent studies have revealed that transfer RNA-derived fragments (tRFs) are closely associated with various diseases; however, whether tRFs contribute to ICP pathogenesis remains unknown. This study was designed with the objectives of investigating the expression profiles of tRFs in patients with ICP, exploring the potential correlation between tRF expression and maternal–fetal pathophysiological changes, identifying novel early diagnostic biomarkers, and ultimately enhancing clinical management strategies.

**Methods:**

Serum samples were collected from 3 ICP patients and 3 healthy controls before delivery. Small RNA sequencing was performed via the Illumina platform, and the obtained sequences were aligned and screened against the tRFdb database to identify differentially expressed tRFs. Potential target genes of tRFs were predicted, followed by Gene Ontology (GO) and Kyoto Encyclopedia of Genes and Genomes (KEGG) pathway enrichment analyses to assess their functional implications.

**Results:**

Compared with controls, ICP patients presented significant differential expression of 5 tRFs, including 2 upregulated tRFs and 3 downregulated tRFs, in prenatal serum. Furthermore, GO and KEGG analyses suggested that fatty acid degradation might be associated with 3003a_trf-3 and 3005b_trf-3.

**Conclusion:**

This study provides preliminary data for the validation of serum-based biomarkers in ICP patients. The findings suggest that tRF dysregulation may be involved in ICP pathogenesis via the fatty acid degradation pathway, offering new molecular insights and a foundation for the development of early intervention strategies to prevent adverse fetal outcomes. These conclusions require further validation in larger sample cohorts.

## 1 Introduction

Intrahepatic cholestasis of pregnancy (ICP) is a common obstetric complication that occurs predominantly in the mid-to-late gestational period, with an incidence rate of 0.2–2% (1). Clinically, ICP is characterized by unexplained pruritus and liver dysfunction. Although symptoms and abnormal biochemical indicators typically resolve spontaneously within 4–6 weeks postpartum and pose minimal risk to the mother, ICP significantly increases fetal complications, e.g., respiratory distress, intrauterine hypoxia, preterm birth, meconium-stained amniotic fluid, and stillbirth (2). The exact etiology of ICP remains incompletely understood, although genetic predisposition, environmental factors, estrogen levels, and immune dysregulation have been implicated in its pathogenesis (3). Owing to the lack of characteristic clinical manifestations in the early stages and the potential for irreversible fetal consequences as the condition progresses, early diagnosis and timely intervention are critical for improving pregnancy outcomes (4).

Transfer RNAs (tRNAs) are a type of small noncoding RNAs (tncRNAs) that decode mRNA codons into amino acid sequences during protein synthesis. tRNA-derived small RNAs (tsRNAs) are a class of single-stranded noncoding RNAs derived from mature tRNAs or tRNA precursors. Based on their cleavage sites (5), they are broadly classified into two major types in human cells: tRNA halves (tiRNAs) and tRNA-derived fragments (tRFs). Although the precise biological functions of tRFs and tiRNAs have not been fully elucidated, accumulating evidence suggests their involvement in gene expression regulation, apoptosis, signal transduction, and epigenetic modifications (6–8).

Emerging studies have linked tRFs and tiRNAs to various diseases. However, their expression patterns and regulatory roles in maternal–fetal systems during pregnancy remain poorly explored.

This study employed small RNA sequencing to profile differentially expressed tRFs between an ICP group and a control group. Subsequently, the differentially expressed genes (DEGs) were evaluated by Gene Ontology (GO) and Kyoto Encyclopedia of Genes and Genomes (KEGG) pathway enrichment analyses to reveal the associated functions. Furthermore, we investigated the potential involvement of tRFs in ICP pathophysiology and explored their utility as novel diagnostic biomarkers to improve clinical management strategies.

## 2 Materials and methods

### 2.1 Study participants

We collected prenatal serum samples from 3 pregnant women diagnosed with ICP (without other comorbidities) (ICP_B_P group) and 3 healthy pregnant women (without complications) as controls (CON_B_P group). Each participant contributed one serum sample, resulting in a total of 6 samples for analysis.

### 2.2 Experimental methods

Serum Sample Collection and Processing: Peripheral blood (4–5 mL) was collected into red-top coagulation tubes (typically in the morning). The samples were processed within 1 h (room temperature) or 2 h (4°C) via centrifugation at 3,000 rpm and 4°C for 10 min. The upper serum layer (1 mL) was carefully aspirated with a pipette, transferred to a 1.5-mL Eppendorf (EP) tube, labeled, and immediately stored at −80°C.

Small RNA Library Preparation and Sequencing: Small RNA libraries were constructed and sequenced from the collected samples.

Sequencing Platform and Parameters: The Illumina HiSeq X 10 system was used in a paired-end (PE150) mode.

## 3 Data processing and analysis

### 3.1 Small RNA sequencing data preprocessing

The raw FASTQ files were processed to remove adapter and primer sequences. Quality control (QC) and length filtering were performed, and only high-quality sequencing fragments were retained. The key filtering step included length selection, with sequences within 15–40 nt (typical range for tsRNAs) retained and those <15 nt or >40 nt discarded. The quality threshold was set at Q20 ≥ 80%. Readings containing ambiguous bases (N) were excluded. Adapter trimming involved the removal of 3′ and 5′ sequencing primer regions from the raw reads. The output metrics included 23.77–32.48 million (M) clean reads per sample. Unique reads were quantified via the fastx_toolkit (v0.0.13). Length distribution analysis of the clean reads and repeat sequence statistics for the unique reads were performed.

### 3.2 tRFs sequence alignment and annotation

To classify and annotate tRFs from the sequencing data, the clean reads were aligned against the tRFdb database ^1^via the following pipeline. Reads with lengths between 15 and 30 nt (characteristic of tRFs) were extracted from the clean_data. Exact-matched alignment (zero mismatches) was performed via Bowtie against the species-specific tRF reference sequences in the tRFdb. The annotated tRFs were categorized into three subtypes: tRF-1 (derived from the precursor tRNA 3′ trailer), tRF-3 (3′ end-derived, including the CCA tail), and tRF-5 (5′ end-derived).

### 3.3 tRFs expression analysis

The expression levels of tRFs were quantified on the basis of their abundance, with higher read counts indicating higher expression. Expression quantification was performed via TPM (transcripts per million) for normalization (9). For differential expression analysis, fold change (FC) values between groups were computed via TPM values. Statistically significant differences were determined by the following criteria: |log_2_FoldChange| > 1 and a *p* < 0.05 (*t*-test). Each tRF was assigned a unique ID in the differential expression list. A volcano plot (significance vs. magnitude of change) and hierarchical clustering heatmap (samplewise expression patterns) were generated.

### 3.4 GO and KEGG pathway enrichment analysis

To elucidate the potential biological functions of the differentially expressed tRFs, we performed target gene prediction, followed by functional enrichment analysis via the following pipeline. Potential tRF target genes were predicted via the miRanda algorithm, which evaluates sequence complementarity and binding energy. For functional annotation, GO analysis was used to categorize the target genes into three domains, namely, biological process (BP), molecular function (MF), and cellular component (CC). KEGG pathway analysis was used to map target genes to KEGG pathways to identify associated metabolic and signaling networks. The results are presented as bar charts (GO term distribution), bubble plots (integrated significance and enrichment scores), and pathway maps (KEGG topology with highlighted genes).

## 4 Results

### 4.1 Differential expression of tRFs in ICP patients vs. controls

**Figure 1:**
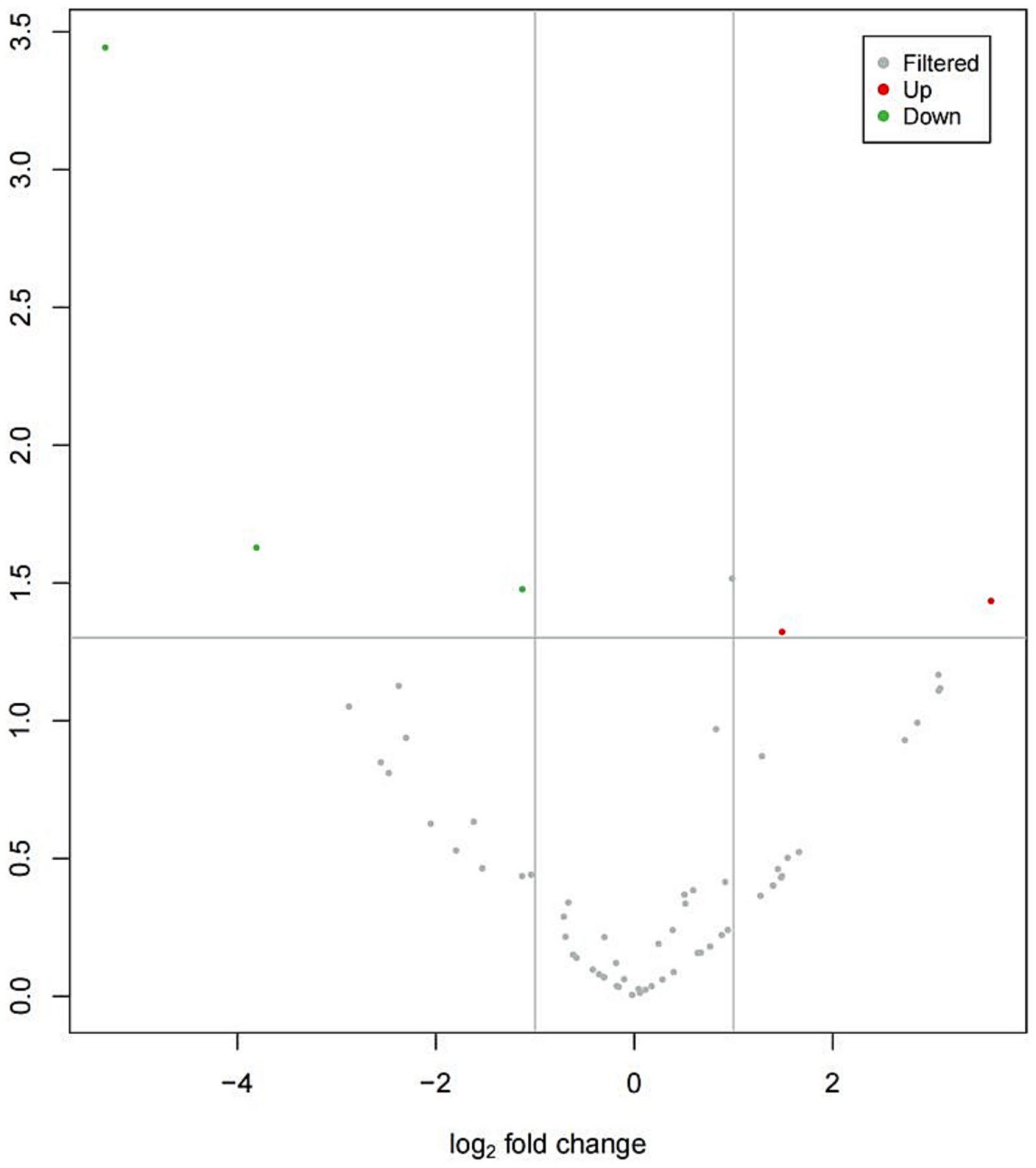
Volcano plot of differentially expressed tRFs in ICP. The volcano plot visualizes tRF expression differences between ICP and control groups:gray points: non-significant tRFs,red points: significantly upregulated tRFs,green points: Significantly downregulated tRFs. X-axis: log2(fold change) values, Y-axis: -log10(*p*-value) for statistical significance.

**Figure 2:**
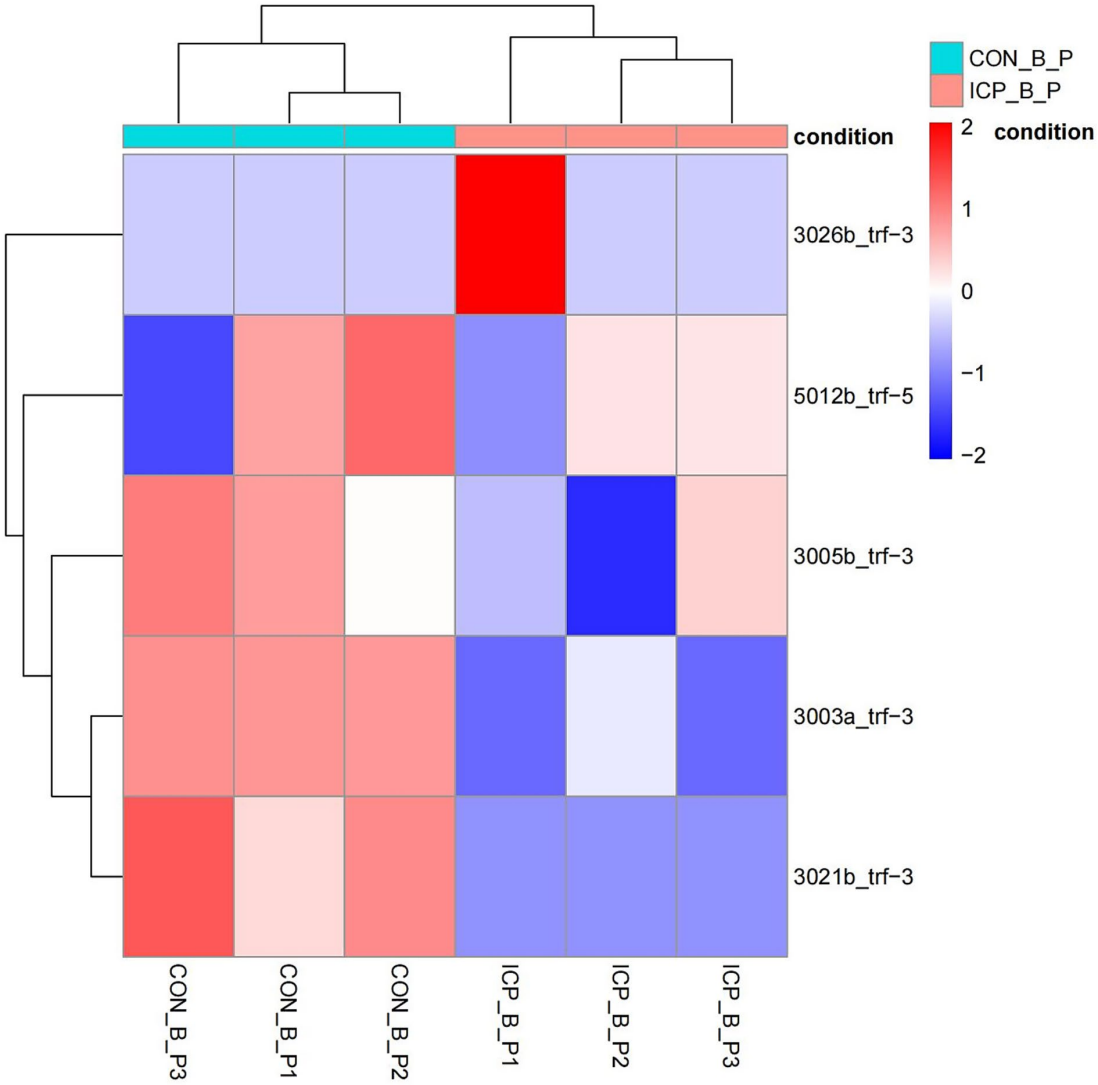
Heatmap of differentially expressed tRFs in ICP. The heatmap displays expression patterns of tRFs meeting the threshold (fold change ≥ 2, *p* < 0.05). Rows: individual tRFs. Columns: biological samples (ICP vs. controls). Red: high expression. Blue: low expression.

Figure 1 provides an overview of tRF expression profiles between the ICP patients and healthy controls. By using the thresholds of |log_2_FoldChange| > 1 and *p* < 0.05, we identified 5 significantly dysregulated tRFs in the ICP group, comprising 2 upregulated tRFs and 3 downregulated tRFs (Table 1 and Figures 1, 2).

**Table 1:** Summary statistics of differentially expressed tRFs.

tRFs_id	log2foldChange	pval	Up_down	Sequence	Length
3003a_trf-3	−5.332	0.000	Down	TCCGGGTGCCCCCTCCA	17
3005b_trf-3	−1.128	0.033	Down	TCAAATCTCGGTGGGACCTCCA	22
3021b_trf-3	−3.809	0.024	Down	TCGATCCCCGGCATCTCCACCA	22
3026b_trf-3	3.596	0.037	Up	TCGATTCCCGGCCAACGCACCA	22
5012b_trf-5	1.490	0.048	Up	GGCTCGTTGGTCTAGGGGTATGA	23

### 4.2 Target gene prediction of differentially expressed tRFs

**Figure 3:**
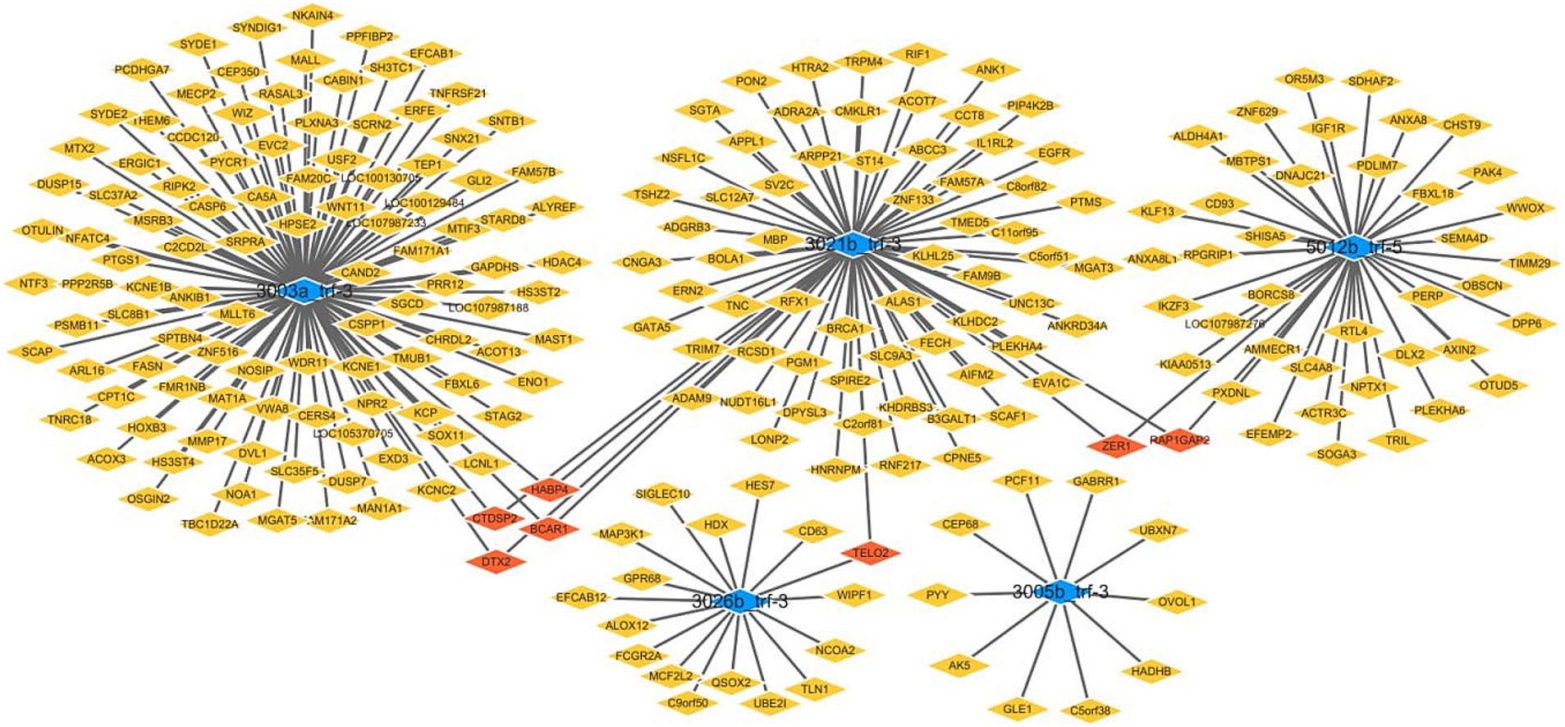
Predicted target genes of differentially expressed tRFs in ICP.

To investigate the potential functional roles of tRFs in ICP pathogenesis, we predicted putative target genes of the dysregulated tRFs via the miRanda algorithm (Figure 3).

### 4.3 GO and KEGG pathway enrichment analysis

**Figure 4:**
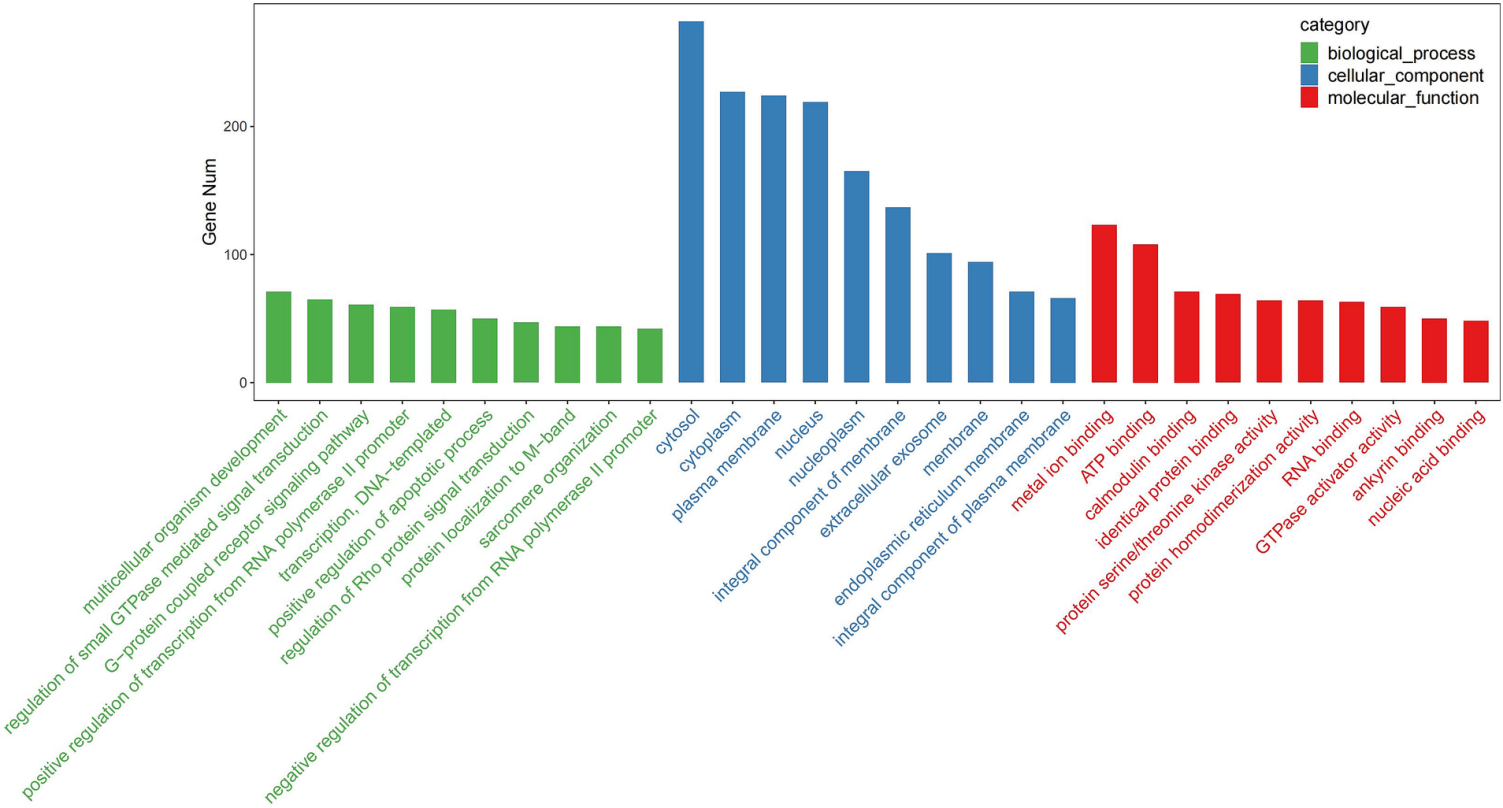
TOP30 GO term distribution. The vertical axis represents the number of enriched genes in each GO term, while the horizontal axis displays the GO term names.

**Figure 5:**
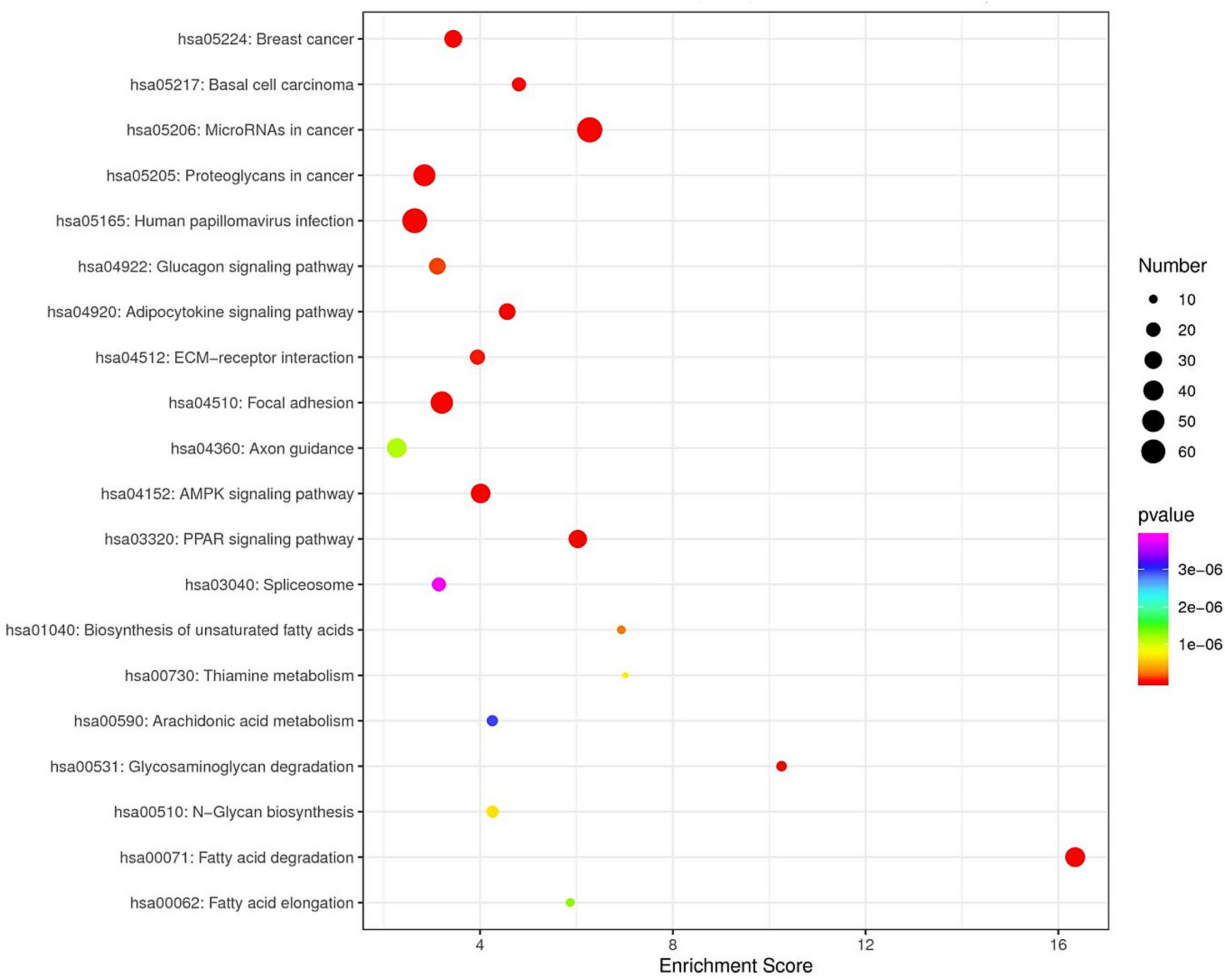
KEGG Enrichment TOP20. In the figure, each dot represents a pathway. Color gradient follows the spectral sequence (red → orange → yellow → green → blue → indigo → violet), corresponding to ascending *p*-values (red = most significant, violet = least significant). Dot size scales with the number of genes in each pathway (larger dots = more genes).

**Figure 6:**
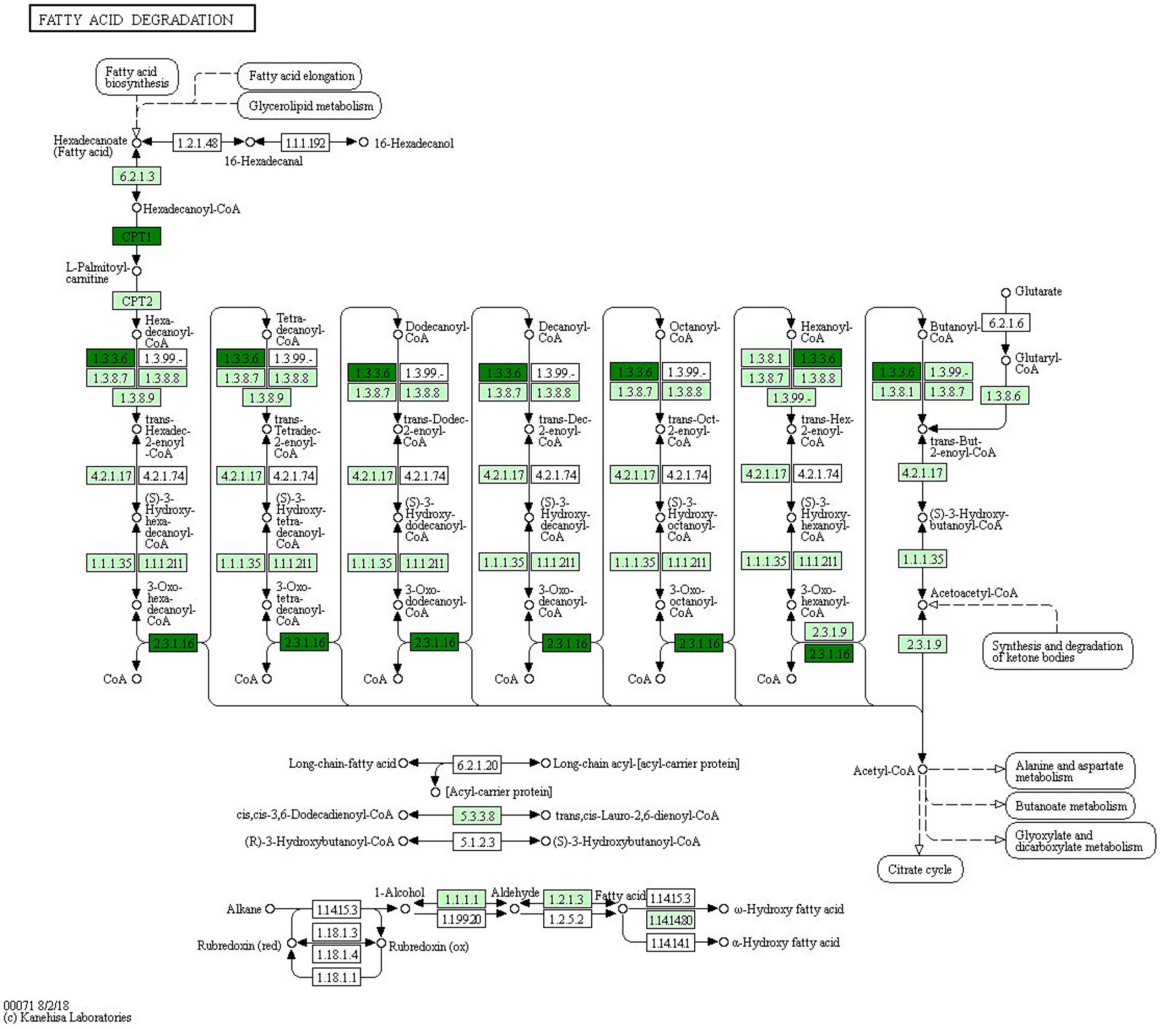
Positioning of the fatty acid degradation signaling pathway. Green indicates downregulated genes. The nodes marked in blackish green (CPT1 and 1.3.3.6) are associated with 3003a_trf-3, the another node marked in blackish green (2.3.1.16) is associated with 3005b_trf-3.

GO enrichment analysis revealed that the predicted target genes of the tRFs were predominantly involved in multicellular organism development among BP terms, metal ion binding and ATP binding among MF terms, and the cytosol among CC terms (Figure 4). Pathway enrichment analysis revealed 20 significantly enriched pathways, among which the fatty acid degradation pathway might play a critical role in the pathogenesis and progression of ICP (Figure 5). In the fatty acid degradation signaling pathway, two downregulated nodes (CPT1 and 1.3.3.6) were associated with 3003a_trf-3, and another downregulated node (2.3.1.16) was associated with 3005b_trf-3 (Figure 6).

## 5 Discussion

tRFs, novel noncoding RNAs, are attracting increasing research attention because of their stability and high expression levels, making them ideal diagnostic and prognostic biomarkers. However, compared with those of classical biomarkers, tRF functions, mechanisms, advantages, and disadvantages remain poorly understood and require further investigation.

ICP is one of the most common complications during the second and third trimesters, and its incidence is influenced by multiple factors (e.g., ethnicity, geographic location, and genetic predisposition) (1). The primary pathological feature of ICP involves excessive accumulation of bile acids in hepatocytes and impaired bile excretion. Elevated serum bile acid levels in pregnant women lead to bile salt deposition in the placental villous space, causing its narrowing and subsequent impairment of maternal–fetal exchange. This pathological process may result in serious complications, e.g., meconium-stained amniotic fluid, fetal distress, preterm birth, and stillbirth (10). While the exact etiology and pathogenesis of ICP remain incompletely understood, genetic factors, environmental influences, estrogen levels, and immune dysfunction have all been implicated in its development. Currently, the most sensitive diagnostic laboratory marker for ICP is elevated serum total bile acid (TBA) levels. Most international consensus guidelines define ICP as TBA levels ≥10 μmol/L, with 40 μmol/L serving as the threshold for distinguishing mild from severe cases (11). The Royal College of Obstetricians and Gynecologists guidelines (12) indicate that abnormal liver function tests and/or elevated bile acid levels are sufficient for ICP diagnosis. However, serum testing shows poor stability, whereas liver function tests, although more reliable, are time consuming and costly. Current diagnostic methods based on clinical symptoms do not allow early ICP detection; therefore, there is an urgent need for auxiliary biomarkers to facilitate early diagnosis, timely treatment, and intervention.

tRFs have been identified across multiple species. Although their precise biological functions remain incompletely characterized, accumulating evidence suggests that they play diverse regulatory roles. tRFs are specific degradation products of tRNAs, and the aberrant expression and functional significance of tRFs in various diseases have attracted considerable research interest (13). Recent advances in sequencing technologies have enabled more comprehensive investigations of tRF regulatory mechanisms. Current studies have demonstrated that tRFs are closely associated with numerous diseases, e.g., cancers and metabolic disorders. Zhang et al. reported altered levels of tRFs in patients with breast cancer (14). Small RNA transcriptome analyses of prostate cancer revealed tRF enrichment in both nonmetastatic and metastatic lymph node samples (15, 16). In addition, animal studies have indicated that sperm tRFs can transgenerationally mediate diet-induced metabolic disorders (17). These significant findings strongly suggest critical roles for tRFs in disease development.

Although current research on tRF expression patterns and regulatory functions during pregnancy is limited, a recent study of maternal immune activation (MIA) in a mouse autism model revealed that tRF expression profiles, similar to those of miRNAs, could distinguish among placental, decidual, fetal brain, and fetal liver tissues. This study demonstrated that tRFs exhibited tissue specificity, developmental variation, and acute responses to environmental stress. These findings suggest potential roles for tRFs in fetal responses to MIA (18).

In our study, we hypothesized that tRFs were differentially expressed and played essential roles in ICP pathogenesis. Our results revealed dysregulated tRF expression profiles in patients with ICP, with 2 upregulated tRFs and 3 downregulated tRFs, suggesting their potential involvement in ICP development. To better understand tRF functions in ICP, we predicted target genes of the differentially expressed tRFs via the miRanda algorithm. Several genes were identified as potential targets, e.g., *CPT1C* (predicted target gene of 3003a_trf-3) and *HADHB* (predicted target gene of 3005b_trf-3). The *CPT1C*-encoded protein regulates beta-oxidation and the transport of long-chain fatty acids into mitochondria and may play a role in the regulation of feeding behavior and whole-body energy homeostasis. *CPT1C* can increase fatty acid utilization, increase cellular ATP levels and preserve redox homeostasis, thereby facilitating cell survival in harsh metabolic environments (19). The *HADHB* gene encodes the beta subunit of the mitochondrial trifunctional protein, which catalyzes the last three steps of the mitochondrial beta-oxidation of long-chain fatty acids. Functional studies have demonstrated that *HADHB* upregulation significantly enhances mitochondrial fatty acid *β*-oxidation (FAO) and reduces intracellular lipid accumulation (20).

On the basis of the GO analysis results, we speculate that abnormal cytoplasmic metal ion and ATP binding-related multicellular organism development processes may be influenced by these differentially expressed tRFs, potentially leading to developmental dysfunction and ICP initiation. The KEGG pathway analysis identified the top 20 enriched pathways, with fatty acid degradation emerging as the most significantly involved pathway. Notably, our experimental results suggested that 3003a_trf-3 and 3005b_trf-3 might be involved in the downregulation of this pathway. Fatty acid metabolism is a complex process, which involves fatty acid uptake and oxidation (21) and can affect endothelial cell function (22). Endothelial cells form the vascular lining and maintain circulatory system homeostasis (23), whereas fatty acid metabolism helps regulate vascular stability (24). Lipid malabsorption during ICP reduces vitamin K absorption, prolongs the prothrombin time and increases the risk of fetal central nervous system (CNS) hemorrhage and perinatal bleeding (3, 25). There is a close mutual regulatory relationship between fatty acid metabolism and bile acid homeostasis, and disruption of this dynamic balance plays an important role in the occurrence and development of various liver diseases. Excessive cholesterol stimulates hepatic bile acid receptors, promoting bile acid synthesis to excrete excess cholesterol, which leads to bile acid accumulation (26, 27). In the event of fatty acid metabolic disorders, the accumulation of free fatty acids in the liver activates inflammatory signaling pathways (e.g., NF-κB), inhibiting farnesoid X receptor (FXR) activity (28, 29). FXR fails to effectively suppress the transcription of cholesterol 7α-hydroxylase (*CYP7A1*), leading to uncontrolled bile acid synthesis and subsequent bile acid accumulation in the liver (30). In diseases such as metabolic-associated fatty liver disease (MAFLD), excessive accumulation of fatty acids in the liver induces lipotoxicity, which inhibits the expression of bile acid transporters, preventing bile acids from being normally excreted from hepatocytes (28, 31, 32). This leads to bile acid accumulation within hepatocytes, exacerbating oxidative stress and hepatocyte apoptosis, thus forming a vicious cycle (27). Elevated TBA levels, which may result from impaired hepatocellular uptake, altered bile acid synthesis, or defective biliary excretion, are a hallmark of ICP (1). Our findings suggest that tRFs, specifically 3003a_trf-3 and 3005b_trf-3, may be associated with the fatty acid degradation pathway. tRFs may impact bile acid metabolism by regulating genes involved in fatty acid oxidation, thereby contributing to ICP pathogenesis. We understand that these suggestions are speculative at this stage and require further experimental validation.

Our study has several limitations. First, although sequencing analysis of three ICP patients initially indicated potential regulatory roles for 3003a_trf-3 and 3005b_trf-3 in ICP, further validation of their expression levels in larger patient cohorts is essential. In this study, the small sample sizes of the ICP and control groups were an intentional preliminary approach. The limited-sample research facilitated the efficient identification of research directions and potential biomarkers, optimizing resource allocation and increasing the effectiveness of subsequent large-cohort studies. Second, we did not experimentally demonstrate the expression and function of ICP-related pathways. Our findings are based solely on tRF sequencing and bioinformatics predictions, and different severities of ICP may influence small RNA sequencing outcomes, as the current sequencing results represent a preliminary screening. Multiple functional experiments are needed to elucidate the regulatory mechanisms of tRFs in ICP pathogenesis. In subsequent experiments, the role of tRFs in the relationship between fatty acid metabolism and bile acid accumulation will be further explored.

## 6 Conclusion

In summary, our pilot study yielded preliminary data that tentatively indicated the possibility that serum-derived tRFs could serve as potential candidate biomarkers for ICP. Through bioinformatics and experimental analyses, we detected significant dysregulation of tRFs (e.g., 3003a_trf-3 and 3005b_trf-3) in ICP patients, which may contribute to disrupted fatty acid degradation. These findings offer novel mechanistic insights into the role of tRF-mediated regulation in hepatic lipid homeostasis and bile acid transport. However, further validation in larger, independent cohorts is needed to confirm the diagnostic utility of these tRFs and their causal relationship with ICP progression. This work establishes a foundational framework for future investigations aimed at developing early intervention strategies to mitigate fetal complications associated with ICP.

## Data Availability

The datasets presented in this study can be found in online repositories. The names of the repository/repositories and accession number(s) can be found in the article/Supplementary material.

## Footnotes

^1^ http://genome.bioch.virginia.edu/search.php
