# Supplementary material for: Dysregulated tRNA-derived fragments impair fatty acid metabolism in intrahepatic cholestasis of pregnancy
Source: Front Med (Lausanne). 2025 Jul 31;12:1630677. doi: 10.3389/fmed.2025.1630677 (PMC12351929; doi:10.3389/fmed.2025.1630677)
Supplement: Supplementary file 1 [file Data_Sheet_1.docx]

Supplementary Material

# Supplementary Data

DOI:10.6084/m9.figshare.29092694
