# Supplementary material for: Dysregulated tRNA-derived fragments impair fatty acid metabolism in intrahepatic cholestasis of pregnancy
Source: Front Med (Lausanne). 2025 Jul 31;12:1630677. doi: 10.3389/fmed.2025.1630677 (PMC12351929; doi:10.3389/fmed.2025.1630677)
Supplement: Supplementary file 2 [file Table_1.docx]

Supplementary Material

# Supplementary Table

**Supplementary Table 1.** Demographic characteristics of participants

| No. | Age^a^ (years) | BMI^a^ (kg/m^2^) | Gestational weeks^a^ | Gravidity | Produce | TBA  (umol/L) |
| --- | --- | --- | --- | --- | --- | --- |
| ICP 1 | 35 | 25.28 | 37 | 2 | 0 | 47.3 |
| ICP 2 | 21 | 21.23 | 38 | 2 | 0 | 43.0 |
| ICP 3 | 28 | 23.53 | 38 | 3 | 1 | 36.7 |
| Con 1 | 24 | 24.02 | 38 | 2 | 1 | 4.9 |
| Con 2 | 32 | 22.66 | 38 | 2 | 1 | 3.5 |
| Con 3 | 31 | 27.34 | 39 | 2 | 1 | 1.9 |

BMI: Body mass index; ICP: Intrahepatic cholestasis of pregnancy;

^a^Two-tailed unpaired Student t-test was preformed to compare the age, BMI, gestational weeks and TBA between two groups. Age: *P* = 0.844; BMI: *P* = 0.506; Gestational weeks: *P* = 0.230; TBA: *P* = 0.000.
